# Crystal Structures of Cif from Bacterial Pathogens *Photorhabdus luminescens* and *Burkholderia pseudomallei*

**DOI:** 10.1371/journal.pone.0005582

**Published:** 2009-05-18

**Authors:** Allister Crow, Paul R. Race, Grégory Jubelin, Carolina Varela Chavez, Jean-Michel Escoubas, Eric Oswald, Mark J. Banfield

**Affiliations:** 1 Department of Biological Chemistry, John Innes Centre, Norwich, United Kingdom; 2 Institute for Cell and Molecular Biosciences, Newcastle University, Newcastle upon Tyne, United Kingdom; 3 INRA, UMR 1225, Toulouse, France; 4 Université de Toulouse, ENVT, UMR 1225, Toulouse, France; 5 INRA, UMR1133 Laboratoire EMIP, Montpellier, France; University of Queensland, Australia

## Abstract

A pre-requisite for bacterial pathogenesis is the successful interaction of a pathogen with a host. One mechanism used by a broad range of Gram negative bacterial pathogens is to deliver effector proteins directly into host cells through a dedicated type III secretion system where they modulate host cell function. The cycle inhibiting factor (Cif) family of effector proteins, identified in a growing number of pathogens that harbour functional type III secretion systems and have a wide host range, arrest the eukaryotic cell cycle. Here, the crystal structures of Cifs from the insect pathogen/nematode symbiont *Photorhabdus luminescens* (a γ-proteobacterium) and human pathogen *Burkholderia pseudomallei* (a β-proteobacterium) are presented. Both of these proteins adopt an overall fold similar to the papain sub-family of cysteine proteases, as originally identified in the structure of a truncated form of Cif from Enteropathogenic *E. coli* (EPEC), despite sharing only limited sequence identity. The structure of an N-terminal region, referred to here as the ‘tail-domain’ (absent in the EPEC Cif structure), suggests a surface likely to be involved in host-cell substrate recognition. The conformation of the Cys-His-Gln catalytic triad is retained, and the essential cysteine is exposed to solvent and addressable by small molecule reagents. These structures and biochemical work contribute to the rapidly expanding literature on Cifs, and direct further studies to better understand the molecular details of the activity of these proteins.

## Introduction

Pathogenic bacteria can subvert host cell function by hijacking vital cellular systems such as the cytoskeleton, vesicular trafficking, apoptosis and the cell cycle [1], [2], [3]. In Gram-negative pathogens, one route to communication with the host involves direct injection of proteins known as ‘effectors’ from the bacterial to host cell cytoplasm using a virulence-associated type III secretion system (T3SS). Following translocation, these effectors function within the host cell, remodelling cellular activities presumably to the benefit of the pathogen.

The host cell cycle is one pathway being increasingly recognised as a target for bacterial virulence mechanisms. Pathogen-derived molecules that modulate the host cell cycle (which have been termed ‘cyclomodulins’) stimulate diverse activities ranging from promotion of cell growth through to altering differentiation and inhibition of cell growth *via* blocking of the cell cycle. The first T3SS-dependent cyclomodulin, known as cycle inhibiting factor (Cif), was initially identified in enteropathogenic *Escherichia coli* (EPEC) and enterohemorrhagic *E. coli* (EHEC) [4]. Using cultured model host cells as a system for infection it was shown that EPEC-Cif (Cif_Ec_) induces re-organisation of the actin cytoskeleton and triggers cell cycle arrests at either the G_2_/M (with associated doubling of DNA content) or G_1_/S phase transitions, in a T3SS-dependent manner [4], [5], [6], [7]. Cif_Ec_ is necessary and sufficient for this activity as an identical phenotype was observed in HeLa cells following delivery of purified recombinant protein using the lipid-based delivery system BioPORTER [4]. Cif-induced cell cycle arrests at both the G_1_ and G_2_ stages are correlated with accumulation of the cyclin-dependent kinase inhibitors (CKIs) p21^waf1/cip1^ and p27^kip1^, which are known to be involved in cell cycle progression [6].

Recently, homologues of Cif have been identified through database searches of other Gram-negative bacteria that, as part of their life-cycle, form pathogenic or symbiotic relationships with a host; each also has at least one T3SS encoded in its genome [8]. These bacteria span different phylogentic classes and include *Burkholderia pseudomallei* (β-proteobacteria), *Yersinia pseudotuberculosis*, *Photorhabdus luminescens* and *Photorhabdus asymbiotica* (all γ-proteobacteria). *B. pseudomallei* is a pathogen of humans and an established infection can cause melioidosis [9]. Infection with *Y. pseudotuberculosis*, also a pathogen of humans, causes gastroenteritis with abdominal pain that can mimic appendicitis [10]. *P. luminescens* is a symbiont for nematodes of the family *Heterorhabditidae* and a pathogen for a broad range of insects [11]. *P. asymbiotica* is an emerging human pathogen [12]. Each of these Cif homologues is capable of inducing cytopathic effects in HeLa cells equivalent to those observed for Cif_Ec_ [8]. Pair-wise alignments with Cif_Ec_ reveal 56%, 26%, 23% and 26% sequence identity for Cifs from *Y. pseudotuberculosis* (Cif_Yp_), *B. pseudomallei* (Cif_Bp_), *P. luminescens* (Cif_Pl_) and *P. asymbiotica* (Cif_Pa_) respectively [8] (Cif_Pl_ and Cif_Bp_ share 36% identity, not counting a 35 amino acid N-terminal extension in Cif_Pl_). Whilst the overall protein sequences diverge significantly, the alignments also reveal strict conservation of residues that comprise the catalytic triad (as identified in the Cif_Ec_ structure) and other residues that are likely important for protein structure and function [8].

The crystal structure of a truncated form of Cif_Ec_ (lacking the N-terminal 99 amino acids) has recently been published [13]. Very recently this has been followed by an independently determined structure of Cif_Bp_ [14]. These structures revealed the presence of a conserved papain-like catalytic triad formed from residues Cys109, His165 and Gln185 (Cif_Ec_ numbering). The spatial arrangement of these residues is very similar to that found in cysteine proteases, and also certain acetyltranferases and transglutaminases. Mutation of the residues that form this triad results in proteins no longer able to induce cytopathic effects in model eukaryotic cells [8], [13], [14]. Details of how Cifs impact the host cell cycle at the molecular level are yet to be determined, and are likely to remain elusive until host cell targets and an observable activity are identified for these proteins.

To further characterise this important family of bacterial cyclomodulins, crystal structures of Cif from *P. luminescens* (Cif_Pl_), and *B. pseudomallei* (Cif_Bp_, independent of that recently published), have been determined. These proteins represent members of the Cif family that are most divergent in sequence from Cif_Ec_. Like Cif_Ec_, these proteins form monomer-dimer equilibria in solution, as does *P. asymbiotica* Cif (Cif_Pa_). The active sites of Cif_Pl_ and Cif_Bp_ are shown to contain reactive cysteines, which are accessible to modification by a thiol-reactive compound. The crystal structures of Cif_Pl_ and Cif_Bp_ both include regions towards the N-terminus of the proteins, referred to as the ‘tail domain’, that were not present in the truncated Cif_Ec_ structure. These domains form an extended surface adjacent to the active site suggestive of a binding interface for interaction with a specific substrate. The positions of the residues comprising the catalytic triad are well conserved, and demonstrate that these proteins are structural as well as functional homologues of Cif_Ec_.

## Materials and Methods

### Protein production

Genes encoding Cif_Ec_, Cif_Bp_, Cif_Pl_ and Cif_Pa_ were all cloned into pET28a (Novagen) for overexpression as described elsewhere [7], [8], with the exception of Cif_Bp_ which was initially cloned to include the amino acid sequence as deposited in UNIPROT (accession number: Q63KH5). The resulting plasmids were transformed into either *E. coli* BL21(DE3) or B834(DE3) strains. Bacterial cultures were grown in Luria-Bertani (LB) media supplemented with kanamycin (50 µg/mL) at 37°C (with shaking) to *A*_600_ between 0.4–0.6 prior to induction of expression with 1 mM isopropyl 1-thio-β-D-galactopyranoside (IPTG). Cultures of B834(DE3) were also supplemented with 50 mg/L L-methionine. Cells were grown for a further 2.5–4 hours before harvesting by centrifugation. For initial preparation of Cif_Bp_, cell pellets were resuspended in 50 mM 4-(2-hydroxyethyl)-1-piperazineethanesulfonic acid (HEPES), 150 mM NaCl, 10 mM imidazole, pH 7.5 supplemented with 5 mM 4-(2-aminoethyl)-benzenesulfonyl fluoride hydrochloride (AEBSF) and lysed by sonication. The lysate was centrifuged and the supernatant applied to pre-equilibrated 5 mL Ni^2+^-IMAC chelating columns (GE Healthcare). The protein was eluted with an imidazole gradient (10–500 mM) over 15 column volumes. Fractions containing protein (as identified by SDS-PAGE) were pooled and concentrated. The protein was then injected onto a Hi-Load 16/60 Superdex 75 column (GE Healthcare) pre-equilibrated with 20 mM trishydroxymethylaminomethane.HCl (Tris), 150 mM NaCl, pH 7.5. For all other preparations (including a clone expressing Cif_Bp_ starting from an alternative start site, see below), cell pellets were re-suspended in 100 mM Tris, 500 mM NaCl, pH 8.2 and lysed using a Constant Systems (UK) T-series high pressure cell disruptor. Clarified lysate was applied to a pre-equilibrated (50 mM Tris, 500 mM NaCl, 5 mM imidazole, pH 8.0) 5 mL Ni^2+^-IMAC chelating column, eluted with a step to 250 mM imidazole in a 7.5 mL volume and loaded on a Hi-Load 26/60 Superdex 75 column (GE Healthcare) pre-equilibrated with 50 mM HEPES, 150 mM NaCl, pH 7.5. Cif proteins elute as a mixture of monomeric and dimeric species with the exception of Cif_Bp_ that appears to run purely as a dimer. Where possible, fractions containing monomer only were pooled, exchanged by ultrafiltration into 20 mM HEPES, pH 7.5 and concentrated to ∼10 mg/mL for further study. Following purification, analysis of Cif_Pl_ by mass spectrometry (matrix-assisted laser desorption/ionisation time of flight (MALDI-TOF)) revealed the protein has a molecular weight of 29,475 Da (compared to theoretical mass of ∼37 kDa (including the vector-derived His-Tag)), suggesting that the protein is cleaved in solution.

For production of selenomethionine-labelled Cif_Pl_ (Cif_Pl_-SeMet) the B834(DE3) *E. coli* strain harbouring the Cif_Pl_-pET28a plasmid was grown initially in LB then diluted into minimal media supplemented with L-methionine and kanamycin as above. At an *A*_600_ of ∼0.6, cells were gently pelleted and re-suspended in minimal media (no methionine) and grown for 10 minutes. 60 mg/L solid L-selenomethionine was then added and the cells grown for a further 20 minutes prior to induction of expression with 1 mM IPTG; cell growth continued at 37°C overnight. Cif_Pl_-SeMet was purified from cell culture as detailed for the native protein.

### Alkylation of reactive site cysteines

The haloalkylating reagent 6 - bromoacetyl - 2 - dimethylaminonaphthalene (badan) reacts with free thiols forming a stable covalent thioether bond. The reaction generates a significant increase in fluorescence intensity on binding. To determine whether Cif proteins contain reactive thiol groups, Cif_Pl_ and Cif_Bp_ were incubated with badan for 1 hour at pH 7.0. The change in fluorescence intensity at 525 nm (excitation at 380 nm) over this time was monitored. The intact mass of the resulting sample was determined by matrix-assisted laser desorption/ionisation time of flight (MALDI-TOF) mass spectrometry. Further, the sites of modification in Cif_Pl_ were mapped by tryptic peptide mass-fingerprinting. MALDI-TOF and mass-fingerprinting analyses were conducted by the Proteomics facility, JIC (Norwich).

### Analytical gel filtration

A Superdex 10/30 GL S200 gel filtration column (GE healthcare) equilibrated with 50 mM HEPES pH 7.2, 150 mM NaCl was used for all analytical gel filtration experiments (flow rate: 0.75 mL/min). Samples were prepared in 250 µL volumes and loaded by syringe into a 100 µL loop before injection onto the column.

### Crystallisation

Crystals of Cif_Pl_ were initially identified using the sitting drop method of vapour diffusion at 20°C in plates set-up with a Douglas Instruments OryxNano crystallization robot and commercially available crystallization screens. The protein solution comprised 12 mg/mL Cif_Pl_ in 20 mM HEPES, pH 7.5. Diffraction quality crystals were grown in 100 mM tri-sodium citrate pH 6.2, 2.45 M ammonium sulfate using 2 µL protein solution mixed with 2 µL precipitant, again in sitting drops. Crystals of the selenomethionine-labelled Cif_Pl_ grew from the same conditions. Crystals grew within three days.

Following analysis of the Cif_Bp_ sequence (see Results) the protein was subjected to a preparative-scale tryptic digest and re-purified on a Hi-Load 16/60 Superdex 75 column, as above. Conditions supporting growth of Cif_Bp_ crystals from this sample were identified using the hanging drop method of vapour diffusion at 20°C and commercially available crystallisation screens using this protein (concentrated to 10 mg/mL). 1 µL protein solution was mixed with 1 µL precipitant solution. After four months single crystals grew from 10% PEG8000, 100 mM HEPES pH 7.5.

### Data collection and structure determination

For data collection, crystals were cryo-protected in either N-paratone oil (Cif_Bp_ and Cif_Pl_-SeMet) or crystallisation buffer containing 22% ethylene glycol (Cif_Pl_) prior to plunging into liquid nitrogen. Diffraction data were collected at either the Daresbury-SRS, station 9.6 (Cif_Bp_) or the Diamond Light Source, stations I03 (Cif_Pl_-SeMet) and I04 (Cif_Pl_). Crystals were maintained at cryogenic temperatures during data collection. All data were processed with MOSFLM [15] and scaled with SCALA [16] (as implemented within the CCP4 suite [17]). 5% of the data were set aside for the calculation of R_free_. A summary of data collection parameters is given in Table 1. The structure of Cif_Pl_ was solved by the single wavelength anomalous dispersion (SAD) method, using the selenomethionine labeled protein crystals. PHENIX.HYSS [18] located all five of the selenium atoms predicted to be in the crystallized protein. These sites were used as input into MLPHARE (as implemented in the CCP4 suite) to obtain initial phases and Hendrickson-Lattman coefficients. Following density modification with DM [19], Arp/Warp [20] was used to build an initial model with ∼150 of 253 residues automatically positioned. This model was refined with REFMAC5 [21] and the resulting maps inspected with COOT [22]. Following initial rebuilding using the SeMet data, refinement was switched to minimize against the isomorphous native dataset. Iterative cycles of refinement and manual rebuilding (using REFMAC5 and COOT) produced the final model which comprises residues Ser50 – Leu302 of the native sequence, 1 sulphate, 1 glycerol and 198 water molecules. A summary of the refinement parameters is given in Table 1.

**Table 1:** Crystallographic data processing and refinement statistics.

	Cif_Pl_ - Native	Cif_Pl_ - SeMet	Cif_Bp_
*Data collection*			
Instrumentation	DLS, station I03	DLS, station I04	Daresbury-SRS, station 9.6
Wavelength (Å)	0.972	0.972	0.92
Space Group	P6_1_22	P6_1_22	P2_1_2_1_2_1_
Resolution range (Å) a	41.96−1.85 (1.95−1.85)	41.83−1.80 (1.90−1.80)	22.01−2.10 (2.21−2.10)
Unit cell parameters (Å)	*a* = *b* = 59.13, *c* = 293.17	*a* = *b* = 58.91, *c* = 292.38	*a* = 54.9, *b* = 77.7, *c* = 115.0
Unique reflections a ^, ^ b ^, ^ d	26,540 (3,343)	29,336 (4,159)	29,440 (4,223)
Multiplicity a ^, ^ b	12.0 (8.4)	10.8 (6.1)	5.0 (4.9)
Mean (I/σ(I)) a ^, ^ d	19.3 (4.4)	23.1 (5.2)	20.2 (3.5)
Completeness (%) a ^, ^ d	97.2 (88.1)	100.0 (100.0)	99.9 (99.9)
*R_sym_* (%) a ^, ^ d	9.5 (38.7)	9.3 (44.6)	5.0 (37.4)
*Refinement*			
*R_cryst_* (%) c	22.6	-	20.9
*R_free_* (%) c	27.4	-	25.9
RmsBond (Å) c	0.010	-	0.017
RmsAngles (°) c	1.254	-	1.607
RmsChiral (°) c	0.094	-	0.111
No. of non-hydrogen atoms	2,233	-	4,008
Cruickshank DPI (Å) c	0.16	-	0.26
Ramachandran outliers	0		0

a Figures in parentheses represent the highest resolution shell.

b Values for multiplicity and completeness of the SeMet dataset are reported for separated anomalous pairs.

c Refinement statistics are as reported by REFMAC version 5.5.0066. RmsBond, RmsChiral and RmsAngle refer to the root-mean-square deviation from ‘ideal’ geometric values described in the Refmac dictionary. *R_free_* = ∑(| |*F*|*_obs_*−|*F*|*_calc_* |)/∑ |*F*|*_obs_* - where |*F*|*_obs_* are observed structure factor amplitudes for a given reflection and |*F*|*_calc_* are corresponding calculated structure factor amplitudes obtained from the refined model. *R_free_* uses only those reflections set aside as a ‘test set’. *R_cryst_* is calculated as per *R_free_* but using the ‘working set’ of reflections.

d Reflection statistics are as reported by SCALA. *R_sym_* is calculated as described in [16].

The structure of Cif_Bp_ was solved using the structure of Cif_Pl_ as a search model. Two molecules were placed in the asymmetric unit by MOLREP. To minimize any bias in the electron density maps, the starting model was converted to a poly-alanine trace and all atomic B-factors were set to 20 Å^2^ before calculation of an initial phase set using REFMAC without refinement. These phases were then used in a density modification procedure involving solvent flattening and two-fold NCS averaging (without recombining the modified and starting phases) using DM. A new model was then built into this ‘low-bias’ electron density map. Further refinement and manual rebuilding (using REFMAC5 (with the NCS restraints gradually loosened and eventually removed) and COOT) produced the final model which comprises residues His14 – Gly262 in chain A, His14 – Leu258 in chain B and 92 water molecules.

For analysis, MOLPROBITY [23] and LSQMAN [24], were used to generate Ramachandran plots and superimposed structures from which root-mean-square deviations (rmsds) based on C_α_ atoms were determined. Protein structure figures have been prepared with PYMOL (DeLano Scientific, USA). The coordinates and structure factors for Cif_Pl_ and Cif_Bp_ have been deposited with the Protein Data Bank with accession codes 3GQJ and 3GQM respectively.

## Results

### Protein production/characterisation

Sequences of the Cif homologues were analysed for regions of disorder using RONN [25]. In Cif_Ec_, the only region of predicted disorder (probability of disorder >50%) is localised to the N-terminal 24 amino acids, which are known to encode the signal for delivery into host cells by the EPEC T3SS machinery [26]. Cif_Pl_, Cif_Bp_ and Cif_Yp_ display more extensive regions of disorder at their N-terminus which includes the first 52, 63 and 47 residues respectively. Only the first 26 amino acids of Cif_Pa_ are predicted to be disordered. Cif_Pl_ is the only member of the family to display disorder at the C-terminus; the last thirteen residues are predicted to be unstructured.

Limited protelysis of Cif_Bp_ with trypsin [27] identified a stable fragment with a molecular weight of ∼30 kDa, as determined from SDS-PAGE gels (not shown). Following scale-up, this fragment was re-purified as described above. MALDI-TOF analysis of this Cif_Bp_ fragment is most consistent with digestion of the protein following Arg55 (PINNACLE Lab., Newcastle University). As a Met residue was observed just downstream of this region (Met61), the possibility that the start-site has been mis-annotated in the sequence database was considered. Re-cloning and functional characterisation of Cif_Bp_ (using delivery of the protein by both the infection model and the BioPORTER system) demonstrates an activity equivalent to the original clone [8]. Therefore, Cif_Bp_ may in fact start at Met61 and amino acid numbering in this study reflects this (i.e. the sequence numbering begins at M(1)ITPIISSNLG).

Following gel filtration, Cif_Pl_ is observed to run at a lower molecular mass than that predicted from the protein's sequence. MALDI-TOF analysis of purified Cif_Pl_ is consistent with cleavage after residue Lys48 of the full-length sequence (assuming no cleavage at the C-terminus; Proteomics Facility, JIC, Norwich). This removes the unstructured region from the N-terminus that was identified bioinformatically, without the addition of exogenous protease.

### Cif proteins adopt monomer-dimer equilibria in solution

Cif_Ec_ can be purified as a monomer or dimer in solution, dependent on the concentration of NaCl [13]. Although it is expected that Cif proteins function as monomers *in vivo*, each of the Cifs produced in this study displays the properties of monomer-dimer equilibria in solution, with the exception of Cif_Bp_ which purifies as a single species (whose apparent molecular weight suggests a dimeric form).

### Alkylation of Cys123 (Cif_Pl_) and Cys90 (Cif_Bp_)

The reactivity of the putative active site cysteines in Cif_Pl_ and Cif_Bp_ was investigated by incubation of the proteins with the halo-alkylating reagent badan. An increase in the fluorescence over time (at 525 nm, not shown) was suggestive of alkylation events. Cif_Pl_ and Cif_Bp_ only have one cysteine in the purified proteins (Cys123 and Cys90 respectively, Cif_Pl_ has a second cysteine in its sequence (Cys20), but this is lost following cleavage of the N-terminal unstructured region). Comparison of the intact mass of the proteins pre- and post-incubation with badan, as determined by MALDI-TOF mass spectrometry, reveals an increase consistent with modification at a single site in both proteins (covalent modification with a single badan molecule would result in a increase of 211 Da). Mass fingerprinting analysis of Cif_Pl_ localises the site of modification to the cysteine-containing peptide ‘IDESVSELGGLEMYQEMVGVNPYDPTEPVCGLSAQNIFK’, which shifts from 4261.5 Da (theoretical mass of 4260.8 Da) to 4472.8 Da (theoretical mass 4471.7 Da), an experimentally measured shift of 211.3 Da. To date, it has not been possible to observe the cysteine-containing peptide in Cif_Bp_ by MALDI-TOF in either the native or modified form.

### Overall structures of Cif_Pl_ and Cif_Bp_

**Figure 1:**
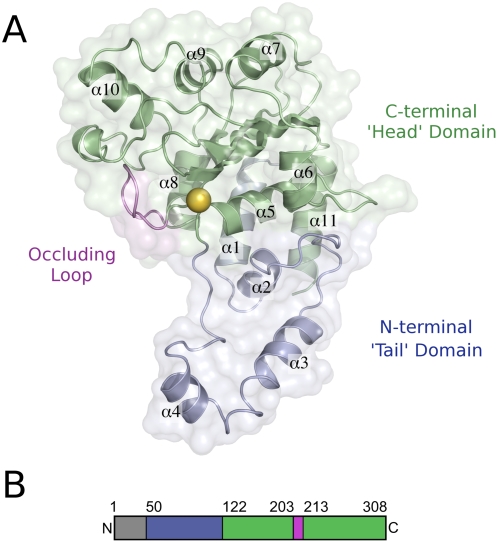
Molecular architecture of Cif_Pl_. (a) The overall structure of Cif_Pl_ resembles a ‘comma’ formed by the tail- and head-domains, which are coloured blue and green respectively. The ‘occluding loop’ is shown in violet, and the location of the proposed active site is marked with a yellow sphere corresponding to the position of the thiol group of Cys123. (b) Domain organisation of Cif_Pl_ as described in the text, coloured as above.

The structures of Cif_Pl_ and Cif_Bp_ closely resemble one another and comprise a head-and-tail domain arrangement reminiscent of a comma or apostrophe (Fig. 1). The structure of Cif_Pl_ overlays on Cif_Bp_ with rmsds of 1.24 Å (chain A, 226 equivalent C_α_ atoms) and 1.25 Å (chain B, 226 equivalent C_α_ atoms). The two chains of Cif_Bp_ overlay with an rmsd of 0.42 Å (245 equivalent C_α_ atoms) and can therefore be considered essentially identical. The tail regions of Cif_Pl_ and Cif_Bp_ are formed by the first 72 and 75 residues of the proteins visible in the structures and are equivalent to a significant proportion of the protein removed in the truncated Cif_Ec_ structure. The head domains of Cif_Pl_ and Cif_Bp_ comprise the C-terminal 60% and 67%, respectively (of the protein visible in the structures), and this region is structurally conserved with Cif_Ec_. Cif_Pl_ and Cif_Bp_ overlay on the structure of Cif_Ec_ (chain A) with an rmsd of 1.33 Å and 1.20 Å respectively (145 and 151 equivalent C_α_ atoms). Therefore Cif_Pl_ and Cif_Bp_, like Cif_Ec_, are structurally related to members of the cysteine protease family and most closely related to AvrPphB [28], a YopT-like effector protein from the plant pathogen *Pseudomonas syringae* pv. phaseolicola. Cif_Pl_ and Cif_Bp_ overlay on AvrPphB with an rmsd of 1.80 Å and 1.76 Å respectively (58 and 56 equivalent C_α_ atoms). The structure of Cif_Bp_ determined here is virtually identical to that recently determined independently [14]. Based on structural homology, Cifs are best classified as papain-like cysteine proteases, although they have not yet been shown to possess protease activity.

### The structure of the active site

A cysteine protease-like catalytic triad was initially identified in the structure of Cif_Ec_ and comprises residues Cys109, His165 and Gln185. In Cif_Pl_ the equivalent residues are Cys123, His181 and Gln200; in Cif_Bp_ they are Cys90, His145 and Gln165. The cysteine of this triad is essential for the activity of Cifs as mutation of this residue leads to loss of the cell cycle arrest phenotype in cell-based assays [8], [14]. The relative positions of the Cys-His-Gln residues in each of the proteins are highly conserved, suggesting these proteins perform the same catalytic function.

**Figure 2:**
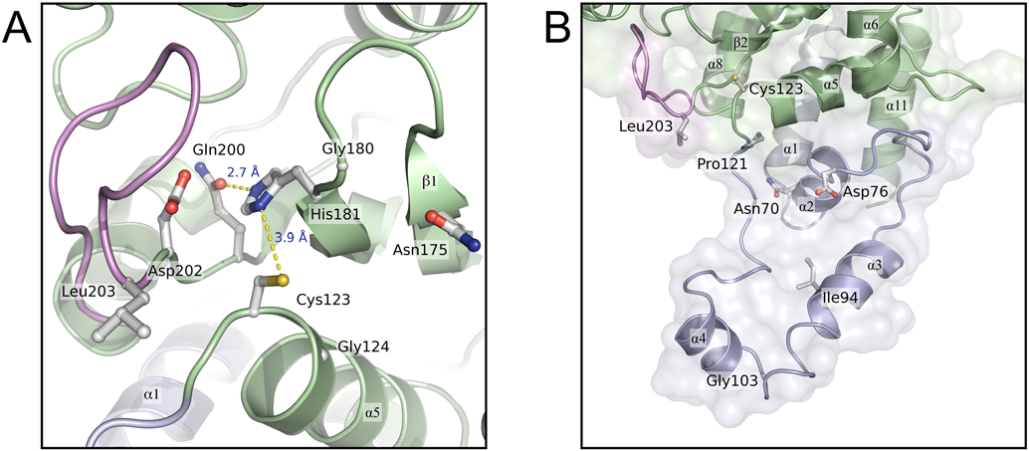
Features of the active site and tail domain of Cif_Pl_. (a) Active site of Cif_Pl_. Hydrogen bonds between residues of the proposed active site triad (Cys123, His181, Gln200) are shown as dotted yellow lines. Residues Gly124, Asn175, Gly180, Asp202 and Leu203, which are fully conserved in Cifs, are also shown. (b) Conserved-residues within the tail-domain. The locations of five residues within the tail-domain that are conserved among all known Cifs are marked. The position of Leu203 and the Cys123 are also shown to aid orientation.

The structure of the active site triad of Cif_Pl_ is shown in Fig. 2(a). The active site cysteine is located at the N-terminus of 13 residue α-helix (α5) with the side chains of His181 and Gln200 arising from the β2 and β3-strands respectively. In both Cif_Pl_ and Cif_Bp_, residues of the catalytic triad are connected through hydrogen bond interactions. The thiol group of the putative catalytic cysteine is positioned 3.9 Å from the N^δ1^ atom of His181 (Cif_Pl_ numbering). The orientation of His181 is stabilised by a hydrogen bond from the N^ε2^ atom of this residue to the O^ε1^ atom of Gln200 (2.7 Å, Cif_Pl_ numbering). These inter-residue distances are similar to those found in papain (which has a Cys-His-Asn triad [29]), and will presumably enhance the nucleophilicity of the active site cysteine in Cifs in a similar manner to that observed in this archetypal cysteine protease. Also, the cysteine thiol group is likely affected by the close approach of the NH groups of backbone amides from residues 124 and 182 (Cif_Pl_ numbering). Further, as noted above, the cysteine residue resides at the N-terminal end of an α-helix. The electrostatic properties of helix macrodipoles have previously been implicated in altering the reactivity (significantly reducing the p*K*_a_) of cysteine residues positioned at the N-termini of α-helices [30].

A number of other residues, conserved across the Cif family, are positioned near the putative active site. The side chain of Asp202 (Cif_Pl_ numbering, see Fig. 2(a)) projects into a solvated pocket in Cifs and could be involved in either modulating the electrostatic properties of the active site or substrate binding. In either role, this residue significantly contributes to the negative surface potential observed at this site (see below). The side chains of Leu203 and Asn175 (Cif_Pl_ numbering) are prominently displayed on the protein surface (see Fig. 2(a)) and are prime candidates for residues that could make interactions with substrates. In addition, glycine residues at positions 124 and 180 (Cif_Pl_ numbering) may be necessary to allow the close-approach of substrates to the active centre. The backbone dihedral angles adopted by these residues are not restricted to glycines, suggesting they are not conserved for purely structural reasons.

### The structure of the occluding loop identified in Cif_Ec_ is conserved in Cif_Pl_ and Cif_Bp_

First observed in the structure of Cif_Ec_, the so-called ‘occluding loop’, which spans residues 189–195 (Cif_Ec_ numbering), lies adjacent to the active site (see Figs. 1 and 2). It has been noted that this loop appears to partially block the catalytic site, and may even regulate access to the active centre via a gating mechanism [13].

The structures presented here show that the conformation of the occluding loop is conserved in both Cif_Pl_ (residues 203–212) and Cif_Bp_ (residues 168–177). Comparison of the B-factors of the residues that comprise the occluding loop with those in the core of the fold suggests the conformation of this region is quite rigid and unlikely to be dynamic. This goes some way to arguing against a role for this loop acting as a flexible gate regulating access to the active site; an alternative role in supporting substrate specificity is discussed below.

### Structure of the tail-domain

As mentioned above, the published structure of Cif_Ec_ is of a truncated form, lacking the first 99 amino acids. The structures of Cif_Pl_ and Cif_Bp_ presented here extend back to residues Ser50 (Cif_Pl_) and His14 (Cif_Bp_). For Cif_Bp_, this directly follows the likely T3SS signal, and it is perhaps not surprising that this is disordered in the structure as it forms the recognition site for the T3SS and/or associated chaperones. For Cif_Pl_, the N-terminal ∼48 residues are cleaved prior to crystallisation, therefore ∼2 residues expected to be in the crystallised protein but are not observed in the electron density.

The overall structure adopted by the tail-domain of Cif_Pl_ is shown in Fig. 2(b). It is formed from four α-helices (in the case of Cif_Pl_: α_1_ = residues 58–71; α_2_ = 72–79; α_3_ = 92–108 and α_4_ = 109–117) and a ‘loop’ region that connects α_4_ to the catalytic cysteine residue at the N-terminus of α5. Structural similarity searches with DALI [31] do not reveal any other proteins (of known structure) that encompass such a domain; this may therefore be unique to Cifs.

**Figure 3:**
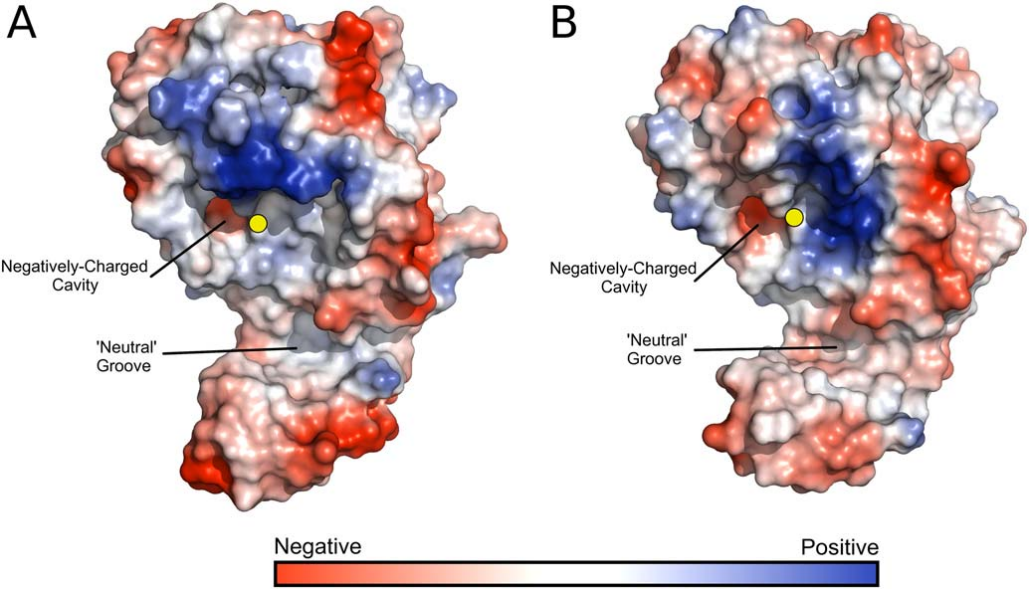
Electrostatic potential surfaces of Cif proteins. (a) Cif_Pl_ (b) Cif_Bp_. Solvent-accessible surfaces were calculated with a 1.2 Å solvent probe radius. Surface colourings are contoured over the same range for each structure (−90 to +90 *k_b_*T/*e_c_*). The location of the catalytic cysteine residue is shown by the yellow sphere.

The region from α_2_ to the active site cysteine forms a prominent surface immediately adjacent to the active site (see Fig. 2(b) and 3) that may be important in determining the substrate specificity for this enzyme. Indeed, mutations within this region in Cifs leads to loss of function in cell-based assays (Oswald, unpublished results, [14]). The tail-domain of Cif_Bp_ (back to residue 14) adopts a conformation very similar to that found in Cif_Pl_, even though there is very little sequence identity between these proteins in this region.

### Surface electrostatics near the active site of Cif_Pl_ and Cif_Bp_

Electrostatic surface representations of Cif_Pl_ and Cif_Bp_ are shown in Fig. 3. The most striking feature is how similar both the overall shape and electrostatic potential of Cif_Pl_ and Cif_Bp_ are, especially close to the active site and within the tail domain. Both proteins contain a patch of positive charge immediately adjacent to the catalytic cysteine and this area may interact with a complementary charged surface on the substrate. Also close to the cysteine is a small cavity displaying negative surface charge. As mentioned above, the largest contributor to the negative surface of this cavity is the side chain of Asp202 (Cif_Pl_ numbering); a similar pocket has been previously noted in Cif_Bp_ [14]. The presence of a neutral groove in the surface is also observed that may have a role in substrate specificity.

### Conserved residues map to the protein surface

**Figure 4:**
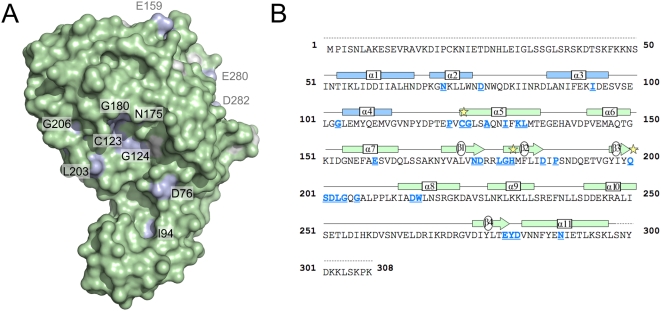
Conserved residues mapped to the surface of Cif. (a) Surface representation of Cif_Pl_ showing locations of solvent-accessible conserved residues. Green are non-conserved, white are semi-conserved and blue are absolutely conserved among all Cif homologues. Residues located on the same face as the active site cysteine are labelled in black, grey labels denote conserved residues that are distant from the active site. (b) Amino acid sequence of Cif_Pl_. Residues that are conserved between all known Cif homologues are marked in blue and underlined. Secondary structure elements are given immediately above the sequence; oblongs and arrows indicate helices and sheets, while a solid line denotes ordered loops. Secondary structure elements are coloured according to the domain structure in Fig. 1. The dashed lines at the N- and C-termini represent regions that are not observed in the crystal structure. Stars indicate residues of the putative active site triad. A sequence alignment for all known Cifs is given in Fig. S1.

A plot of the surface of Cif_Pl_ coloured according to sequence conservation across the protein family reveals conserved, surface accessible residues cluster in two locations (Fig. 4(a)). The first of these includes the active site and surrounds. Potential roles for residues Gly124, Asn175, Gly180, Asp202 and Leu203 have been described above. Asp76 and Ile94 reside within the tail domain. While Ile94 is partially exposed, it is largely buried in the hydrophobic core of the tail domain and likely stabilises its structure. Asp76 is prominently displayed projecting towards the catalytic site. As the side chain has no obvious role in maintaining the architecture of the protein fold, this residue may be involved in interacting with substrate. The second location includes residues Glu159, Glu280 and Asp282. This region is distant from the active site, and the role of this conserved surface patch is not immediately apparent.

## Discussion

Gram negative bacterial pathogens harbouring a virulence associated type III secretion system (T3SS) are able to directly inject effector proteins into host cells to modulate their activity, presumably to the benefit of the pathogen. One family of T3S effectors, the Cifs, re-program the host cell cycle causing arrest at the G_2_/M or the G_1_/S transition. Significant advances in understanding the molecular basis of Cif activity have recently been made with the demonstration of conserved activity in cell-based assays for all known Cif family members and structural studies.

In this study the crystal structures of Cif from *P. luminescences* (Cif_Pl_) and *B. pseudomallei* (Cif_Bp_) are presented. The structures reveal a head-and-tail domain arrangement, with the head-domain adopting an overall fold similar to papain-like cysteine proteases. The structure of the tail-domain of Cif_Pl_ and Cif_Bp_ presents an extensive surface area adjacent to the active site suggestive of a binding surface for interaction with a specific substrate (see Fig. 2(b), 3 and 4). Despite recent advances in the studies of Cifs, the specific activity encoded within the proteins (e.g. protease/acetyltransferase) and the specific host cell target/s remain to be determined. This has, perhaps, been more challenging than expected and one explanation for this is that Cifs may be highly specific for their *in vivo* substrate. Due to its position near the active site, a significant contribution to this specificity will likely be conferred by the tail-domain, but will not be limited to this. As mentioned above, it has been suggested that the presence of an occluding loop in Cifs may restrict access to the active site cysteine residue. However, incubation of Cif_Pl_ and Cif_Bp_ with the halo-alkylating reagent badan reveals this residue in Cif_Pl_ and Cif_Bp_ is addressable in solution. A similar result has also recently been observed for Cif_Ec_ and Cif_Bp_ using the protease inhibitor E-64 (*trans*-epoxysuccinyl-l-leucylamido(4-guanidino)butane) [14]. In the structures of Cif_Pl_ and Cif_Bp_, 24.0% and 25.7% of the surface area of the cysteine sulphur atoms respectively are exposed to solvent (28.1 Å^2^ and 30.1 Å^2^, calculated using AREAIMOL (as implemented in CCP4 [17]) with a 1.2 Å probe radius). Although badan is only a small molecule, not another protein (the likely host cell target of Cifs), this shows that no accessory factors are required to enable access to the cysteine from solution. Therefore, rather than blocking access to the active site, the occluding loop may be involved in binding substrate/determining substrate specificity.

A set of 31 amino acid residues are fully conserved in all five Cif sequences known to date (see Fig. 4(b) and S1). 27 of these were present in the truncated Cif_Ec_ structure and these largely cluster at three locations: (1) the active site and surrounding region, (2) a region that may be important for maintaining structural integrity and (3) a region for which sequence/structural conservation was not immediately apparent from the Cif_Ec_ structure [8]. The structures of Cif_Pl_ and Cif_Bp_ presented here show that the overall position of these 27 residues are spatially conserved in Cifs. Residues clustering in the active site region (Pro121, Gly124, Ala127, Asn175, Leu179-Gly180, Ser201-Gly204, Gly206, Asp215-Trp216 (Cif_Pl_ numbering)) are expected to be either essential for maintaining the relative positions of the triad residues or be involved in substrate binding. A role for the second region (including residues Asp186, Asp188 and Glu280-Asp282 (all Cif_Pl_ numbering)) in maintaining a side-chain mediated inter-strand hydrogen bond has been suggested [8]; this region also contains residues that comprise the second conserved surface patch (Fig. 4(a)), as mentioned above. A function for the third region (residues Lys132, Leu133 and Asn289, Cif_Pl_ numbering) is, however, revealed in the structures of Cif_Pl_ and Cif_Bp_. In these structures these residues contribute to a surface that the loop between α_2_ and α_3_ (part of the tail-domain) engages with. Variation at these positions would destabilise the docking of this region onto the rest of the structure.

The structures of Cif_Pl_ and Cif_Bp_ also present an opportunity to put the remaining four conserved residues into a structural context: Asn70, Asp76, Ile94 and Gly103 (Cif_Pl_ numbering, Asn36/Asp42/Ile60/Gly69 in Cif_Bp_ and Asn52/Asp58/Ile76/Gly88 in Cif_Ec_). Firstly, the O^δ1^ atom of Asn70/36 forms a hydrogen bond to the backbone nitrogen of Pro118/Leu85 (Cif_Pl_/Cif_Bp_ numbering respectively), anchoring the loop between α_4_ and α_5_ (which contains the active site cysteine residue at its N-terminus). A stable, conserved structure in this region will be crucial for the integrity of the catalytic centre and, likely, for recruiting substrate. Putative roles for Asp76/42 and Ile94/60 have been described in the Results section. Gly103/69 resides within a tight turn between α_3_ and α_4_ (see Fig. 2(b)), and the importance of this residue is likely to be structural.

While Cif proteins are expected to function as monomers *in vivo*, all Cifs recombinantly expressed to date form either monomer-dimer equilibria in solution or are predominantly dimeric, as revealed by gel filtration. However, crystal packing in the structures of Cif_Pl_ and Cif_Bp_ are not consistent with a conserved oligomeric state across the protein family, and any significance of the dimeric forms observed in solution is not immediately apparent. Interestingly, a dimeric state was also observed in the structure of the truncated Cif_Ec_ [13]. The structures of Cif_Pl_ and Cif_Bp_ presented here show that this ‘helix-swapped’ dimer cannot be consistent with the dimer formed in solution by the full-length proteins as the tail-domain will fill the space occupied by the C-terminal helix from the second molecule in this structure.

When delivered to cultured model host cells by an active T3SS, or via BioPORTER, Cif proteins induce the same phenotype, suggesting they recognise and act on a common substrate. For this to be the case, the surface properties in the region of the active site should be similar. Analysis of the electrostatic surface of Cif_Pl_ and Cif_Bp_ (Fig. 3) and the localisation of conserved residues (labelled black in Fig. 4(a)) in this region is supportive of this conclusion. Further analysis of any specific interaction now requires identification of the *in vivo* substrate of Cifs, and the role of these conserved residues will be the subject of future work.

In summary, this manuscript describes the structures of Cif_Pl_ and Cif_Bp_, building on previous structural and functional studies of this family of bacterial proteins that arrest the eukaryotic cell cycle. While the specific substrate upon which these proteins act is yet to be determined, these structures identify intriguing putative interacting surfaces whose function can be investigated by mutagenesis. The papain-like fold and putative catalytic triad originally identified in the structure of Cif_Ec_ are well conserved in Cif_Pl_ and Cif_Bp_, suggesting a conserved function for the protein family.

## Supporting Information

Figure S1 Alignment of known Cif sequences.
(0.02 MB TIF)

## References

[1] Galan JE, Wolf-Watz H (2006). Protein delivery into eukaryotic cells by type III secretion machines.. Nature.

[2] Stebbins CE (2005). Structural microbiology at the pathogen-host interface.. Cell Microbiol.

[3] Stebbins CE, Galan JE (2001). Structural mimicry in bacterial virulence.. Nature.

[4] Marches O, Ledger TN, Boury M, Ohara M, Tu X (2003). Enteropathogenic and enterohaemorrhagic Escherichia coli deliver a novel effector called Cif, which blocks cell cycle G2/M transition.. Mol Microbiol.

[5] Nougayrede JP, Boury M, Tasca C, Marches O, Milon A (2001). Type III secretion-dependent cell cycle block caused in HeLa cells by enteropathogenic Escherichia coli O103.. Infect Immun.

[6] Samba-Louaka A, Nougayrede JP, Watrin C, Jubelin G, Oswald E (2008). Bacterial cyclomodulin Cif blocks the host cell cycle by stabilizing the cyclin-dependent kinase inhibitors p21 and p27.. Cell Microbiol.

[7] Taieb F, Nougayrede JP, Watrin C, Samba-Louaka A, Oswald E (2006). Escherichia coli cyclomodulin Cif induces G2 arrest of the host cell cycle without activation of the DNA-damage checkpoint-signalling pathway.. Cell Microbiol.

[8] Jubelin G, Chavez CV, Taieb F, Banfield MJ, Samba-Louaka A (2009). Cycle inhibiting factors (CIFs) are a growing family of functional cyclomodulins present in invertebrate and mammal bacterial pathogens.. PLoS ONE.

[9] Wiersinga WJ, van der Poll T, White NJ, Day NP, Peacock SJ (2006). Melioidosis: insights into the pathogenicity of Burkholderia pseudomallei.. Nat Rev Microbiol.

[10] Naktin J, Beavis KG (1999). Yersinia enterocolitica and Yersinia pseudotuberculosis.. Clin Lab Med.

[11] Boemare NE, Akhurst RJ, Mourant RG (1993). DNA relatedness between Xenorhabdus spp. (Enterobacteriaceae), symbiotic bacteria of entomopathogenic nematodes, and a proposal to transfer Xenorhabdus luminescens to a new genus, Photorhabdus gen. nov.. Int J Syst Bacteriol.

[12] Gerrard J, Waterfield N, Vohra R, ffrench-Constant R (2004). Human infection with Photorhabdus asymbiotica: an emerging bacterial pathogen.. Microbes Infect.

[13] Hsu Y, Jubelin G, Taieb F, Nougayrede JP, Oswald E (2008). Structure of the cyclomodulin Cif from pathogenic Escherichia coli.. J Mol Biol.

[14] Yao Q, Cui J, Zhu Y, Wang G, Hu L (2009). A bacterial type III effector family uses the papain-like hydrolytic activity to arrest the host cell cycle.. Proc Natl Acad Sci U S A.

[15] Leslie AG (2006). The integration of macromolecular diffraction data.. Acta Crystallogr D Biol Crystallogr.

[16] Evans P (2006). Scaling and assessment of data quality.. Acta Crystallogr D Biol Crystallogr.

[17] Collaborative Computational Project 4 (1994). The CCP4 suite: programs for protein crystallography.. Acta Crystallogr D Biol Crystallogr.

[18] Adams PD, Grosse-Kunstleve RW, Hung LW, Ioerger TR, McCoy AJ (2002). PHENIX: building new software for automated crystallographic structure determination.. Acta Crystallogr D Biol Crystallogr.

[19] Cowtan KD, Zhang KY (1999). Density modification for macromolecular phase improvement.. Prog Biophys Mol Biol.

[20] Morris RJ, Perrakis A, Lamzin VS (2003). ARP/wARP and automatic interpretation of protein electron density maps.. Methods Enzymol.

[21] Murshudov GN, Vagin AA, Dodson EJ (1997). Refinement of macromolecular structures by the maximum-likelihood method.. Acta Crystallogr D Biol Crystallogr.

[22] Emsley P, Cowtan K (2004). Coot: model-building tools for molecular graphics.. Acta Crystallogr D Biol Crystallogr.

[23] Davis IW, Leaver-Fay A, Chen VB, Block JN, Kapral GJ (2007). MolProbity: all-atom contacts and structure validation for proteins and nucleic acids.. Nucleic Acids Res.

[24] Kleywegt GJ, Zou JY, Kjeldgaard M, Jones TA, Rossmann MGaA, E. (2001). International Tables for Crystallography, Vol. F. Crystallography of Biological Macromolecules;.

[25] Yang ZR, Thomson R, McNeil P, Esnouf RM (2005). RONN: the bio-basis function neural network technique applied to the detection of natively disordered regions in proteins.. Bioinformatics.

[26] Charpentier X, Oswald E (2004). Identification of the secretion and translocation domain of the enteropathogenic and enterohemorrhagic Escherichia coli effector Cif, using TEM-1 beta-lactamase as a new fluorescence-based reporter.. J Bacteriol.

[27] Reece RJ, Maxwell A (1991). Probing the limits of the DNA breakage-reunion domain of the Escherichia coli DNA gyrase A protein.. J Biol Chem.

[28] Zhu M, Shao F, Innes RW, Dixon JE, Xu Z (2004). The crystal structure of Pseudomonas avirulence protein AvrPphB: a papain-like fold with a distinct substrate-binding site.. Proc Natl Acad Sci U S A.

[29] Kamphuis IG, Kalk KH, Swarte MB, Drenth J (1984). Structure of papain refined at 1.65 A resolution.. J Mol Biol.

[30] Miranda JJ (2003). Position-dependent interactions between cysteine residues and the helix dipole.. Protein Sci.

[31] Holm L, Kaariainen S, Rosenstrom P, Schenkel A (2008). Searching protein structure databases with DaliLite v.3.. Bioinformatics.

